# Performance comparison of CareStart™ HRP2/pLDH combo rapid malaria test with light microscopy in north-western Tigray, Ethiopia: a cross-sectional study

**DOI:** 10.1186/s12879-017-2503-9

**Published:** 2017-06-06

**Authors:** Daniel Getacher Feleke, Shambel Tarko, Haftom Hadush

**Affiliations:** 1 Department of Biomedical Sciences, College of Health Sciences and Referral Hospital, Aksum University, Aksum, Ethiopia; 2 School of Nursing, College of Health Sciences and Referral Hospital, Aksum University, Aksum, Ethiopia

**Keywords:** Malaria, RDT, Light microscopy, North-western Tigray

## Abstract

**Background:**

Rapid diagnostic tests (RDTs) are alternative methods for microscopy in the diagnosis of malaria in resource limited settings. Among commercially available RDTs, CareStart™ Malaria test was found to show reliable results. This study evaluated the performance of CareStart™ Malaria Combo test kit in Northwestern Tigray in Ethiopia.

**Methods:**

Blood samples were collected from 320 malaria-suspected patients at Mayani Hospital in Northwestern Tigray from December 2015 to March 2016. All blood samples were examined using both light microscopy and CareStart™ Malaria HRP2/pLDH Combo Test kit. Statistical analyses were performed using SPSS version 20.

**Results:**

The overall parasite positivity using light microscopy and CareStart™ RDT was 41 (12.8%) and 43 (13.4%), respectively. The sensitivity and specificity of CareStart™ RDT, regardless of species, were found to be 95.4 and 99.3%, respectively. Furthermore, the sensitivity of CareStart™ RDT for *Plasmodium falciparum* or mixed infection and non-*falciparum* malaria parasites was 94.4 and 85.0%, respectively while the specificity was found to be 98.9 and 99.7%, respectively. The agreement between the two test methods was “excellent” with a kappa value of 0.92.

**Conclusion:**

CareStart™ RDT has very good sensitivity and specificity for malaria diagnosis. The test kit also has an excellent agreement with light microscopy. It is therefore useful in resource-limited areas where microscopy is not available.

## Background

With an estimated 214 million cases worldwide, and 438, 000 deaths annually, malaria still causes high morbidity and mortality [1]. In Ethiopia, approximately 75% of the total area is estimated to be “malarious” with about 68% (52 million people) of the total population being at risk of infection [2, 3]. Malaria transmission is unstable and seasonal in most parts of the country due to altitude and climatic factors [4, 5]. It is responsible for 10–40% of patient consultation in Health institutions throughout the country [6]. *Plasmodium falciparum* and *Plasmodium vivax* are the dominant Plasmodium species with 60–70% and 30–40% reported cases, respectively [4]. Currently, the main strategy for malaria control is quick and accurate diagnosis followed by effective treatment [7, 8]. Among the diagnosis methods, microscopy is considered the “gold standard” because it has high sensitivity, inexpensive to perform, allows Plasmodium species identification and quantification of parasite density [9, 10]. Unavailability of well-trained laboratory personnel and other resource limitations to perform microscopic test makes RDTs a good alternative method for malaria diagnosis in resource poor settings [9–11]. However, RDTs cannot quantify the parasitic load and are not effective to diagnose recently treated individuals due to the persistence of malarial proteins in the blood stream after treatment [10].

RDTs are based on the detection of histidine-rich protein 2 (HRP-2), plasmodium specific lactate dehydrogenase (pLDH) and aldolase antigens [12, 13]. Among the RDTs, the three-band CareStart™ malaria test which detects HRP-2 and PAN-pLDH had shown good results for the diagnosis of different Plasmodium species [5]. It is based on lateral flow immunochromatography in cassette format which can detect *P. falciparum*-specific protein and pan-*Plasmodium* protein. It fulfills the performance criteria set for the rapid diagnosis of malaria by attaining sensitivity of greater than 95% for samples with parasitemia ≥ 100 parasites/μl of blood [10]. As a result, CareStart™ Malaria HRP-2/ pLDH (Pf/PAN) combo test has been widely used by health extension workers at health posts in Ethiopia since 2005 [10]. Many studies were conducted to evaluate the performance of RDTs in different geographical areas [9, 10].

In Ethiopia, CareStart™ Malaria Combo test was evaluated in Afar, Kola Debi Health Center and Southern Ethiopia. In these studies the overall sensitivity and specificity of CareStart™ malaria combo test was 94.2–98.5% [5, 10, 14]. CareStart™ Malaria Combo test kit is among the widely used RDT kits in areas where microscopy is not feasible. The evaluation of RDT kits’ performance in different geographical areas and at different level of parasite densities has a paramount importance. This study aimed to evaluate the performance of CareStart™ Malaria Combo test kit in Northwestern Tigray, Ethiopia.

## Methods

### Study area and participants

**Fig. 1:**
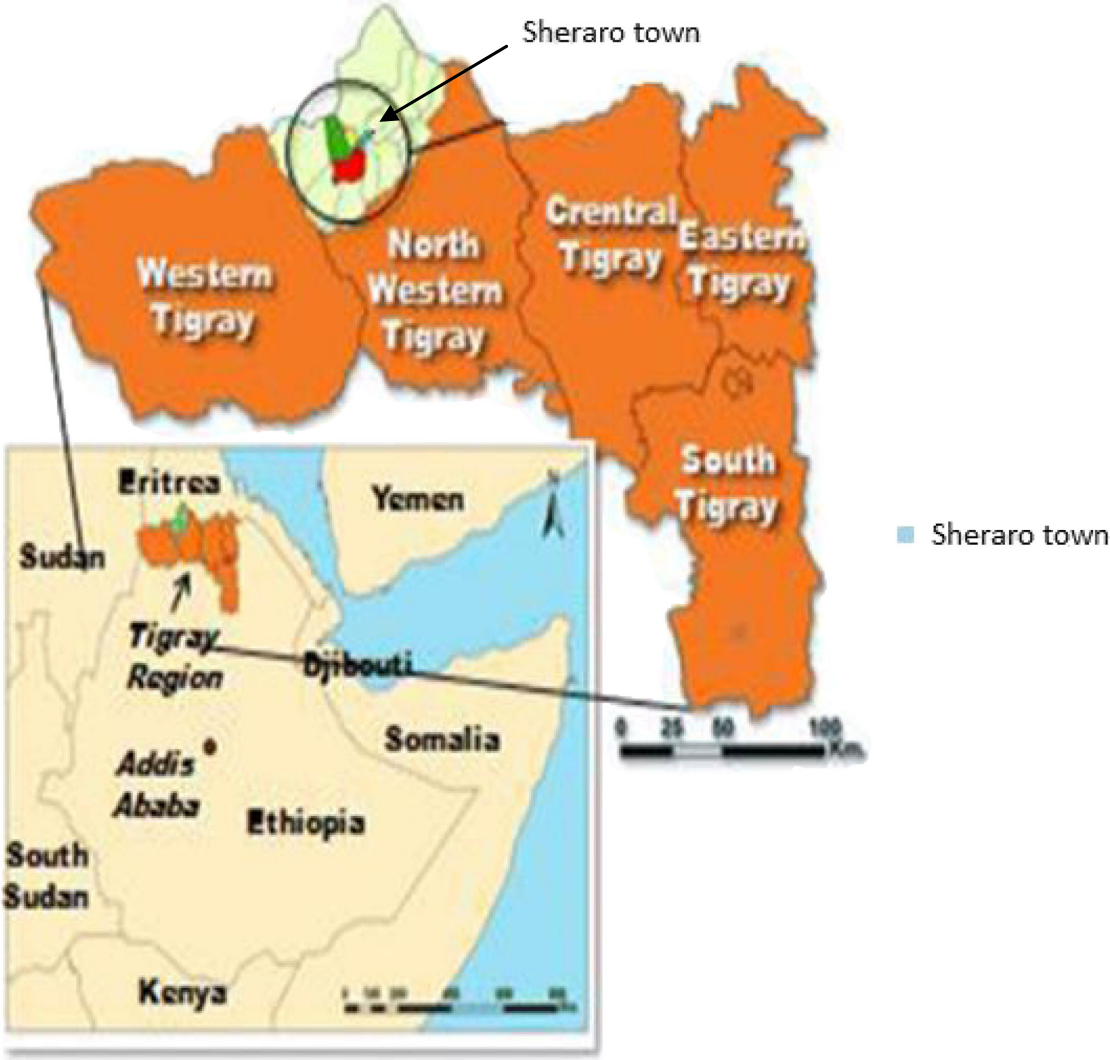
Location of Sheraro town Northwestern Tigray, Ethiopia [22]

This cross-sectional study was carried out in Mayani Hospital Northwestern Tigray from December 2015 to March 2016. Mayani Hospital is located in Sheraro town; Sheraro is a town and separate district in Northern Ethiopia (Fig. 1). It is located in the Tigray region, a place with an elevation of 1246 m above sea level. The town is located about 1117 km north of Addis Ababa, the capital of Ethiopia and 402 km Northwest of Mekelle, the capital of the regional state. Sheraro is one of the malaria endemic areas in western Tigray. The town has a total population of 17,045 among which 8163 are men and 8882 women.

Mayani Hospital is located 5 km away from the town and provides service for communities around the town. All malaria-suspected symptomatic patients at Mayani Hospital were included in the study. Malaria-suspected patients are those who manifest signs and symptoms of malaria such as fever, headache, fatigue and sweating/chills. Mayani Hospital is preferred by the community due to its quality health service and fair price. As a result, it has high malaria-suspected patient load in Northwestern Tigray. Three- hundred and twenty acute febrile illness patients suspected of malaria were eligible in this study. Patients who have been treated with anti-malarial drugs in the past 4 weeks from time of sample collection and those who were in serious health conditions were excluded. Demographic information was collected using a questionnaire. Each malaria-suspected patient was tested using both microscopy and CareStart™HRP2/pLDH combo rapid malaria test and results were recorded in pre-designed spread sheet.

### Sample size determination

Sample size was calculated using single-proportion sample size formula [15].

$$ \mathrm{n}={{\mathrm{Z}}^2}^{\ast}\mathrm{p}\ \left(1-\mathrm{p}\right)/{\mathrm{d}}^2 $$

Where: P = the prevalence of malaria = 23.8% N = sample size,

Z = 95% confidence interval (1.96), d = Margin of error (5%);

N = (1.96)^2^*0.238(1–0.238)/ (0.05)^2^

N = 3.8146*0.18/0.0025

N = 276.7 = 277 However, to enhance the reliability of the test, an additional 43 more malaria-suspected patients were recruited.

### Specimen collection and processing

Two (2) ml of venous blood sample was collected using EDTA-anticoagulated tube for the detection of malaria parasite using both microscopy and CareStart™HRP2/pLDH Combo rapid malaria test methods. Two experienced laboratory technologists were assigned in the study hospital to collect blood sample, prepare blood film and perform RDT. Staining and microscopic examination of blood films were performed in Aksum University Referral Hospital diagnostic laboratory by another two experienced microscopists who had training and are certified in malaria microscopy. They were unaware about CareStart™HRP2/pLDH Combo rapid malaria test results. The discordant between the two microscopists was examined by a third highly experienced expert microscopist and the result was taken as final. Microscopy and RDT performers had training before the start of the study. In addition, the investigators were in close contact with data collectors to assess the quality of data throughout the data collection period. To assess the consistency of microscopic examination, 30 blood smears were examined blindly by a laboratory technologist who was not part of the study. He detected malaria parasites in three of the blood smears previously reported as negative. However, only one of the three blood smears was positive by the experienced expert microscopist.

### Microscopic examination

Thin and thick blood smears were prepared from each clinically-suspected malaria patients. Blood films were sent to Aksum University Referral Hospital in a standard slide box for staining with 3% Giemsa and microscopic examination. Thin smears were considered positive for malaria if one or more malarial parasites were seen; and, negative if no asexual form of *Plasmodium* was observed in 200 high-power fields. On the other hand, thick blood films were taken as positive if one or more malaria parasites have been observed; and, negative if no parasites were seen after examining 1000 white blood cells [14].

### Rapid diagnostic tests

CareStart™HRP2/pLDH Combo rapid malaria test (manufactured by Access BIO, Inc. Monmouth junction New Jersey 08852) was based on lateral flow immunochromatography in cassette format which can detect *Plasmodium falciparum*-specific protein and pan-*Plasmodium* protein. The storage temperatures of the kits were between 4 and 30 °c.

Five (5) μl blood samples and diluents were added on the kit and reading was performed at 20 min according to the manufacturer’s directions. Results were recorded as negative (no test line visible) or positive (at least one test line visible). If the test line was barely visible, the result was scored as *doubtful* and repeated. If the control line did not appear, the test was considered *invalid* and was repeated [16]. All RDT results were read by laboratory technologists who had experience in performing RDT and were blinded to the microscopy test results.

Microscopically-confirmed and malaria-free blood samples were used as a positive and negative control respectively to check the validity of CareStart™ RDTs.

### Data analysis

The data were entered in Microsoft Excel spreadsheet, exported and analyzed by SPSS version 20. Two-way contingency table analysis and cross tabulations were used to show the association and odds ratio (OR) of study population characteristics and parasite positivity by light microscope. Sensitivity, specificity and predictive values for the detection of different Plasmodium species were calculated and compared against each other. Kappa value was determined to evaluate the agreement between CareStart™ RDT and light microscopy. A *p*-value of less than 0.05 was considered as significant in all comparisons.

### Ethical approval

Consent form was used to ask patients’ or guardians’ (in case of children) willingness. Ethical clearance was obtained from the institutional review board (IRB) of Aksum University, College of Health Sciences. Microscopically-confirmed malaria positive study participants were treated with appropriate anti-malaria drug in the study hospital.

## Results

A total of 320 suspected malaria patients were tested using light microscopy and the CarStart™ malaria RDT. The male-to-female ratio was 1.3:1. The age range of study participants was from 6 to 53 years and majority of them (166 (51.9%)) were between 15 and 45 years age group. Most of the study participants 227 (70.9%) were from the rural areas of the district. The age groups 15–45 (OR = 2.2, 95% CI = 1.0–4.3) and above 45 years old (OR = 2.1, 95% CI = 1.0–4.1) were more likely to be malaria positive using microscopy (Table 1).

**Table 1:** Parasite positivity as detected by light microscope at different socio-demographic characteristics of patients in Northwestern Tigray, Ethiopia, December 2015 to March 2016

Socio-demographic characteristics of study participants		Number tested (%)	Number positive (%)	Number negative (%)	OR	95% CI
Sex	Male	181 (56.5)	20 (11.0)	161 (89.0)	1.4	0.7–2.8
	Female	139 (43.4)	21 (15.1)	118 (84.9)		
Age	<15	68 (21.3)	10 (14.7)	58 (85.3)	2.0	1.0–4.1
	15–45	166 (51.9)	14 (8.4)	152 (91.6)	2.1	1.0–4.3
	>45	86 (26.9)	17 (19.8)	69 (80.2)	2.1	1.0–4.1
Residence	Rural	227 (70.9)	193 (85)	34 (15.0)	0.5	0.2–1.1
	Urban	93 (29.1)	86 (92.5)	7 (7.5)		

The overall parasite positivity using light microscopy was 41 (12.8%): 16 (5.0%) for *P. falciparum*, 20 (6.2%) for *P. vivax* and 5 (1.6%) for mixed infections. While using the CareStart™ RDT, the overall parasite positivity was 43 (13.1%): 19 (5.6%) for *P. falciparum*, 19 (5.9%) for *P. vivax* and 5 (1.6%) for mixed infections (Table 2).

**Table 2:** Malaria parasite positivity based on light microscopy and CareStart™RDT

		CareStart RDT				
		Negative	*P.falciparum* Number (%)	*P.vivax* (PAN only) Number (%)	Mixed (pf + PAN) Number (%)	Total
Light microscope	Negative	276 (86.3)	2 (0.6)	1 (0.3)	0 (0.0)	279 (87.2)
	*P. falciparum*	0 (0.0)	15 (4.7)	1 (0.3)	0 (0.0)	16 (5.0)
	*P. vivax*	1 (0.3)	0 (0.0)	17 (5.3)	2 (0.6)	20 (6.3)
	Mixed	0 (0.0)	2 (0.6)	0 (0.0)	3 (0.9)	5 (1.6)
Total		277 (86.6)	19 (5.9)	19 (5.9)	5 (1.6)	320 (100.0)

The sensitivity and specificity of light microscopy for *P. falciparum* or mixed infection was compared with that of RDT. Whereas, the sensitivity, specificity and predictive values of light microscopy for non-*falciparum* infections was compared to the “PAN only” CareStart RDT results. The sensitivity of the CareStart™ RDT for *P. falciparum* or mixed infection and non-*falciparum* malaria parasites was 94.4 and 85%, respectively. The overall sensitivity and specificity of CareStart™ RDT was found to be 95.35 and 99.29%, respectively. The positive predictive value (PPV) and negative predictive value (NPV) were 99.6 and 98.92% respectively. The agreement between the CareStart™ RDT and light microscopy tests was excellent with a kappa value of 0.92 (Table 3). There was also a very good agreement between the two test methods in detecting different Plasmodium species.

**Table 3:** Performance of CareStart™ by species identified in comparison to the standard reference light microscopy

	Pf or mixed by LM vs pf/pan by RDT	Non-Pf (by LM) vs Pan only (by RDT)	All positive by LM vs all positive by RDT
Sensitivity (%) (95% CI)	94.4 (94.3–94.5)	85.0 (84.8–85.2)	95.4 (95.3–96.0)
Specificity (%) (95% CI)	98.9 (98.8–99.0)	99.7 (99.5–100.0)	99.3 (99.2–99.4)
Positive predictive value (%) (95% CI)	85.0 (84.8–85.14)	89.5 (89.4–89.6)	99.6 (99.58–99.62)
Negative predictive value (%) (95% CI)	99.0 (98.9–99.04)	99.3 (99.2–99.4)	98.9 (89.8–90.0)
Agreement between tests Kappa	0.85 (0.76–0.91)	0.89 (0.78–0.93)	0.92 (0.80–0.93)

*Pf Plasmodium falciparum*, *LM* light microscopy, *RDT* rapid diagnostic test

The parasitic density of each *P. falciparum* and *P. vivax* positive sample was counted and calculated using the standard formula for parasitemia. Three samples had parasite count of <100/μl of blood (Table 4).

**Table 4:** Over all sensitivity of CareStart™ HRP2/PLDH Combo Test for the detection of Plasmodium species at different levels of parasitemia in North-Western Tigray, Ethiopia, December 2015 to March 2016

No of parasite/μl of blood)	Plasmodium species	Microscopy (n)	CareStart™ (n)
		41	43
>4000		N	N
1001–4000	*P.falciparum*	4	3
	*P. vivax*	5	6
501–1000	*P.falciparum*	7	8
	*P. vivax*	6	5
100–500	*P.falciparum*	5	7
	*P. vivax*	8	6
	Mixed	5	5
<100	*P. vivax*	1	2
	*P.falciparum*	0	1

*N* none, *n* number, *P. falciparum Plasmodium falciparum*, *P. vivax Plasmodium vivax*

## Discussion

Malaria is one of the major public health problems in Ethiopia where ¾ of the landmass is malarious and an estimated 68% of the population lives in these areas [2, 3]. The CareStart™ Malaria HRP-2/ pLDH (Pf/PAN) combo test has been widely used by health extension workers at health posts in Ethiopia since 2005 [10]. In the present study, CareStart™ RDT showed high sensitivity and specificity (Table 3). The high sensitivity and specificity of CareStart™ RDT was in line with a report from Kola Diba Health Center, Northwest Ethiopia [14, 17]. This study revealed a lower sensitivity and higher specificity compared to reports from Northeast Ethiopia and Sierra Leone [2, 5]. Generally, CareStartTM RDT showed good sensitivity and specificity as compared to that of light microscopy.

The diagnostic performance of the CareStart™ RDT for the diagnosis of different Plasmodium species was evaluated. When compared to light microscopy, CareStart™ RDT falsely detected *P. falciparum* infection in two of the blood samples. These false positive results might be due to the persistence of HRP-2 in blood stream. Whereas, the false-positive test results for *P. vivax* might have occurred due to the high levels of *P. falciparum* parasite in circulation [18]. Despite the clearance of the asexual parasite forms, pLDH test may remain positive because of pLDH production by plasmodial gametocytes which may result in false positive result [19]. This assumption was in line with the detection of two *P. falciparum* and one *P. vivax* gametocytes in the present study.

The false negative results might be due to low parasitemia as a result of sequestration which can reduce the number of parasites in circulation below the detectable level [10]. In this study, the parasite density of *P. vivax* was below 100/ μl of blood which might be responsible for the false negative results by CareStart™ RDT.

The current study showed higher sensitivity and specificity for *P. falciparum* or mixed infection than Moges et al.*’s* report [14]. The sensitivity of CareStart™ RDT for the detection of *P. falciparum* and *P. vivax* was lower than a report from Wondo Genet, Southern Ethiopia by Sharew et al. [16]. In contrast to Heutmekers M et al. report from a retrospective evaluation of CareStart™ RDT [20], the sensitivity and specificity for *non-P. falciparum* parasite detection were higher. Similarly, the overall sensitivity of CareStart™ RDT for the detection of *P. falciparum* and *P. vivax* was higher than Jessica Maltha et al. report [21]. This might be due to the detectable parasite density in the present study as only three positive samples had the parasite density of below 100/ μl of blood. The sensitivity of RDTs increased as the level of parasites in circulation higher.

The high negative and positive predictive values of CareStart™ RDT in the present study was in agreement with a study conducted in Kola Diba Health Center, Northwest Ethiopia [14]. This indicates the ability of RDT was very high to correctly diagnose malaria negative and positive patients.

As detected by either of the CareStart™ RDT (13.4%) or the light microscope (12.8%), the overall prevalence of malaria in this study was very low compared to Moges et al. report (40.9%) [14]. The low prevalence could be partly due to the fact that the data was collected just after the end of major malaria transmission season in the country. Another possible reason might be due to the effectiveness of malaria prevention and control strategies in Ethiopia. The main strategies were awareness creation, increased bed net distribution and budget increment in malarious areas of the country. In contrast to Moges et al. report, CareStart™ RDT showed more parasite positivity than light microscopy. This might be partly due to the late clearance of circulating plasmodium proteins in blood.

## Conclusion and recommendation

CareStart™ RDT has very good sensitivity and specificity for malaria diagnosis. It has also an excellent agreement with light microscopy. Therefore, continued use of this RDT is highly recommended in resource-limited areas where microscopy is not available.

## Abbreviations

HRP-2: Histidine rich protein-2
NPV: Negative Predictive value
pLDH: plasmodium lactate dehydrogenase
PPV: Positive Predictive value
RDT: Rapid Diagnostic Test
